# Early immune surveillance to predict cytomegalovirus outcomes after allogeneic hematopoietic stem cell transplantation

**DOI:** 10.3389/fcimb.2022.954420

**Published:** 2022-08-03

**Authors:** Jintao Xia, Xuejie Li, Genyong Gui, Jian Wu, Shengnan Gong, Yuxin Shang, Jun Fan

**Affiliations:** ^1^ State Key Laboratory for Diagnosis and Treatment of Infectious Diseases, Collaborative Innovation Center for Diagnosis and Treatment of Infectious Diseases, The First Affiliated Hospital, College of Medicine, Zhejiang University, Hangzhou, China; ^2^ Department of Life Sciences, Faculty of Natural Sciences, Imperial College London South Kensington Campus, London, United Kingdom

**Keywords:** human cytomegalovirus, hematopoietic stem cell transplantation, immune indices, HCMV IgG, lymphocyte, enzyme-linked immunospot assay

## Abstract

**Background:**

There is no method of predicting human cytomegalovirus (HCMV) outcomes in allogeneic hematopoietic stem cell transplant recipients clinically, leading in some cases to excessive or insufficient antiviral therapy. We evaluated the early immune response of recipients with disparate HCMV outcomes.

**Methods:**

The HCMV outcomes of recipients were determined by long-term monitoring of HCMV DNA levels posttransplant. HCMV IgG and IgM concentrations at 1 week before and 1 week after transplantation, absolute lymphocyte counts, and HCMV-specific IFN-γ secreting cells at 1 month posttransplant were evaluated based on HCMV outcome.

**Results:**

All recipients were negative for HCMV IgM. Significant differences between recipients with and without HCMV reactivation were observed in pre- and post-transplant HCMV IgG antibody levels, absolute lymphocyte counts, and HCMV-specific IFN-γ secreting cells (P < 0.05). HCMV IgG antibody levels significantly increased after transplantation in recipients with HCMV reactivation (P = 0.032), but not in those without reactivation. Multivariate analysis revealed that except for the absolute lymphocyte count these biomarkers were related to HCMV reactivation, independent of other clinical factors. In time-to-event analyses, lower levels of these biomarkers were associated with an increased 150-day cumulative incidence of HCMV reactivation (log-rank P < 0.05). In recipients with HCMV reactivation, the duration of HCMV DNAemia had negative correlation with HCMV-specific IFN-γ-secreting cells (P = 0.015, r = -0.372). The relationships between the peak HCMV DNA load and absolute lymphocyte count and HCMV-specific IFN-γ-secreting cells followed the same trends (P = 0.026, r = -0.181 and P = 0.010, r = -0.317).

**Conclusions:**

HCMV IgG, absolute lymphocyte count, and HCMV-specific IFN-γ secreting cells represent the humoral and cellular immune response. Early monitoring of these immune markers could enable prediction of HCMV outcomes posttransplant and assessment of the severity of HCMV DNAemia.

## Introduction

Human cytomegalovirus (HCMV) seroprevalence is high worldwide (Manicklal et al., 2013; Zuhair et al., 2019), and HCMV reactivation is a frequent issue in recipients of allogeneic hematopoietic stem cell transplantation (allo-HSCT) (Kaito et al., 2020), in whom it causes direct and indirect harmful effects (Hancock et al., 2020; Mori et al., 2020).

Antivirals are used for HCMV in most bone marrow transplantation centers, but are costly and can cause myelosuppression, organic lesions, and emergence of drug-resistant viruses (Bregante et al., 2000; Marty et al., 2020). The inadequacy of the standard clinical diagnosis and treatment results in overtreatment of patients, bringing a risk for drug toxicity, while undertreating others, who remain at high risk for HCMV and may experience other adverse consequences or aggravated HCMV infection posttransplant (Camargo et al., 2018). Thus, it is imperative to have an early estimate of HCMV outcomes.

Early immune indices can facilitate assessment of immune responses. Three biomarkers that reflect humoral and cellular immune responses were used in this study because of their role in controlling HCMV reactivation and the feasibility of measuring them in clinical laboratories (Paez-Vega et al., 2018; Yoon et al., 2021). These early immune indices showed significant differences between individuals with and without HCMV reactivation, and correlations with the duration of HCMV DNAemia and peak viral loads in recipients with HCMV reactivation.

## Materials and methods

### Subjects

In total, 221 HCMV-seropositive recipients who had undergone allo-HSCT in the First Affiliated Hospital, College of Medicine, Zhejiang University between March 2018 and May 2021 were selected. The exclusion criteria were age < 18 years; infection with hepatitis B virus, Treponema pallidum, or human immunodeficiency virus; other cancers; infusion of chimeric antigen receptor T cells; autologous HSCT; and retransplantation. Antiviral prophylaxis and preemptive therapy were performed according to hospital transplant procedures.

Detection of HCMV DNA by real-time quantitative fluorescence polymerase chain reaction (qPCR) is recommended to guide the initiation of, and monitor the response to, preemptive therapy (Ljungman et al., 2019). Based on the HCMV DNA load posttransplant, the subjects were divided into three subgroups: NCR, recipients who experienced no HCMV reactivation; OCR, recipients who experienced one episode of HCMV reactivation; and RCR, recipients who experienced recurrent HCMV reactivation, defined as two or more episodes (separated by at least two negative HCMV DNA results). HCRs represented recipients who experienced HCMV reactivation, which included OCRs and RCRs.

The study was approved by the Ethics Committee of the First Affiliated Hospital, College of Medicine, Zhejiang University, and was conducted in accordance with the Declaration of Helsinki.

### Samples

Ethylene diamine tetraacetic acid (EDTA) anticoagulant and coagulation-promoting peripheral blood samples were collected before and after transplantation. Serum was separated from samples taken at 1 week before and 1 week after transplantation. Peripheral blood mononuclear cells (PBMCs) were prospectively prepared on post-HSCT day 30 by Ficoll centrifugation and cryopreserved in freezing medium (90% fetal bovine serum and 10% dimethyl sulfoxide) according to the manufacturer’s instructions (Cedarlane, Australia).

### Monitoring of HCMV DNA load

HCMV DNA load was tested at 1-week intervals in the first 3 months after transplantation and monthly or bimonthly thereafter using Nucleic Acid Detection Kits (Daan, Zhongshan, China). Briefly, HCMV DNA was extracted from fresh whole-blood samples and subjected to qPCR analysis. HCMV reactivation was defined as detection by qPCR of HCMV DNA in whole blood at ≥ 500 copies/mL. The initial and peak HCMV DNA loads were defined as the first detectable and the highest DNA levels recorded posttransplant, respectively.

### HCMV IgG/IgM detection

Serum HCMV IgG/IgM levels were determined using an indirect/antigen-capture enzyme-linked immunosorbent assay (ELISA) according to the manufacturer’s instructions (DIA.PRO Diagnostic Bioprobes, Italy).

### Absolute lymphocyte count

Absolute lymphocyte count (ALC) at day 30 after allo-HSCT, as determined using an Automatic Blood Cell Analyzer (BC5390, Mindray, China), was obtained from the laboratory informatics system.

### HCMV-specific IFN-γ secreting cells

The IFN-γ enzyme-linked immunospot (ELISpot) assay was performed as previously described (Zhang et al., 2019) with slight modification. Briefly, in the experimental group, 2×10^5^/well PBMCs were seeded on a 96-well plate precoated with anti-IFN-γ antibody (IFN-γ ELISpot Assay Kits; Mabtech, Sweden), and stimulated with 15-amino-acid (11-amino-acid overlap) peptides that spanned the HCMV pp65 protein (Miltenyi, Germany). RPMI 1640 medium and mAb-CD3–2 were added to the negative and positive controls, respectively, as stimulants. The cells were then incubated at 37°C in an incubator equilibrated with 5% CO_2_ for 30 h. Captured IFN-γ was labelled after incubation for 2 h at room temperature by adding ALP-conjugated secondary antibody. Next, filtered substrate solution was added to induce spot development. Finally, spots were counted using an automated ImmunoSpot S5 Versa Analyzer (Cellular Technology Ltd., Shaker Heights, OH). PBMCs that secreted IFN-γ when stimulated with the pp65 peptide pool were described as IFN-γ-secreting cells (ISCs).

HCMV pp65 is the dominant antigen that elicits an HCMV-related immune response (Abate et al., 2004), and such peptide pools have been used to provide insight into cellular immunity (Dodero et al., 2009).

### Statistical analysis

Descriptive statistics of demographic and clinical characteristics were calculated. The nonparametric Mann–Whitney U-test and Kruskal–Wallis test were used to compare groups of continuous variables. Categorical variables were compared using a chi-square test or Fisher’s exact test, as appropriate. For correlation analyses, the Spearman’s rank test was used. Optimal biomarker cutoffs were identified using X-tile software (Camp et al., 2004). Univariate analyses were performed using Cox proportional hazards regression models. Multivariate models were created using variables with a P < 0.05 in the univariate models, together with those considered clinically relevant. Differences in HCMV reactivation probability between groups were subjected to a log-rank test. P < 0.05 was considered indicative of statistical significance. Statistical analysis and visualization were performed using IBM SPSS 26.0 (IBM SPSS Statistics), Prism version 7.0 (GraphPad Software Inc., San Diego, CA) and R version 3.6.2 (R Foundation for Statistical Computing, Vienna, Austria).

## Results

### Recipients’ characteristics

**Figure 1:**
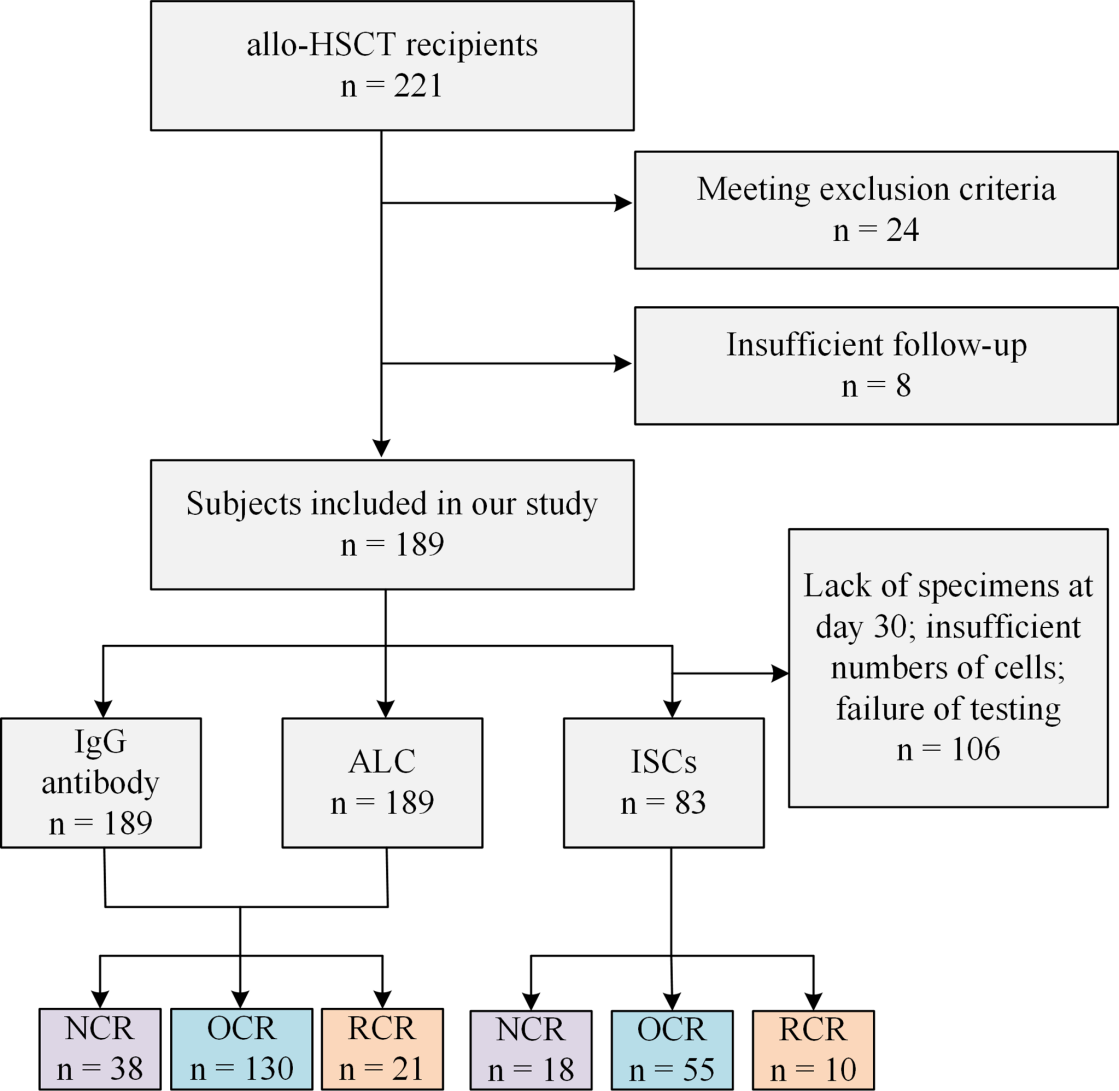
Study participant flowchart. allo-HSCT, allogeneic hematopoietic stem cell transplant; ALC, absolute lymphocyte count; ISCs, HCMV specific IFN-γ secreting cells; NCR, recipients without HCMV reactivation; OCR, recipients with one episode of HCMV reactivation; RCR, recipients with recurrent HCMV reactivation.

The recipients and samples analyzed are shown in **Figure 1**, and the recipients’ characteristics are listed in **Table 1**. There were no significant differences in baseline characteristics among the three groups, except that the proportion of males was significantly lower in the NCR group than in the OCR and RCR groups (**Table 1**).

**Table 1:** Patient characteristics and clinical outcomes.

Characteristic	All patients	NCR (n=38)	OCR (n=130)	RCR (n=21)	*P* ^$^	*P* ^&^
**Age, median (IQR), y**	41 (31-51)	41 (32-51.5)	40 (29-51)	47 (37-53)	0.16	0.07
**Gender**						
Male	97 (51.3)	12 (31.6)	72 (55.4)	13 (61.9)	0.021*	0.641
Female	92 (48.7)	26 (68.4)	58 (44.6)	8 (38.1)		
**D(onor)/R(ecipient)** **HCMV serostatus**						
D^+^ R^+^	189	38	130	21	1.000	1.000
**Underlying disease**						
Acute myeloid leukemia	88 (46.5)	14 (36.8)	65 (50)	9 (42.9)	0.340	0.641
Acute lymphoblastic leukemia	48 (25.4)	11 (29)	30 (23.1)	7 (33.3)	0.513	0.411
Chronic leukemia	7 (3.7)	1 (2.6)	6 (4.6)	0	0.850	1.000
Lymphoma	9 (4.8)	3 (7.9)	6 (4.6)	0	0.486	1.000
MDS/MPN	27 (14.3)	8 (21.1)	16 (12.3)	3 (14.3)	0.357	0.730
Other	10 (5.3)	1 (2.6)	7 (5.4)	2 (9.5)	0.457	0.613
**Conditioning regimen**						
AraC + BuCy + MECCNU + ATG	118 (62.4)	14 (36.8)	89 (68.5)	15 (71.4)	0.001*	1.000
AraC + BuCy + MECCNU	4 (2.1)	3 (7.9)	1 (0.75)	0	0.054	1.000
AraC + VP-16 + BuCy + MECCNU	9 (4.8)	6 (15.8)	3 (2.3)	0	0.007*	1.000
AraC + VP-16 + BuCy + MECCNU + ATG	2 (1.1)	0	1 (0.75)	1 (4.8)	0.250	0.260
BuCy + MECCNU + ATG	16 (8.5)	4 (10.5)	11 (8.45)	1 (4.8)	0.844	1.000
BuCy + MECCNU	15 (7.9)	7 (18.5)	7 (5.4)	1 (4.8)	0.042*	1.000
Flu + BU + ATG	16 (8.5)	4 (10.5)	11 (8.5)	1 (4.8)	0.844	1.000
CLAG + AraC + BuCy + MECCNU + ATG	6 (3.2)	0	4 (3.1)	2 (9.5)	0.126	0.196
DAC + AraC + BuCy + MECCNU + ATG	1 (0.5)	0	1 (0.75)	0	1.000	1.000
BuCy + ATG	2 (1.1)	0	2 (1.5)	0	1.000	1.000
**ATG usage**	161 (85.2)	22 (57.9)	119 (91.5)	20 (95.2)	<0.001*	0.701
**Immunosuppressive agents**						
MTX + CsA + MMF	177 (93.7)	29 (76.3)	127 (97.7)	21 (100)	<0.001*	1.000
MTX + CsA	12 (6.3)	9 (23.7)	3 (2.3)	0		
**Stem cell source**						
Peripheral blood	189 (100)	38 (100)	130 (100)	21 (100)	1.000	1.000
**Type of donor**						
Total-matched related	30 (15.9)	18 (47.4)	11 (8.45)	1 (4.8)	<0.001*	1.000
Half-match related	134 (70.9)	17 (44.7)	99 (76.15)	18 (85.7)	<0.001*	0.410
Unrelated	25 (13.2)	3 (7.9)	20 (15.4)	2 (9.5)	0.502	0.740
**aGVHD**	51 (27)	3 (7.9)	40 (30.8)	8 (38.1)	0.009*	0.614
**Steroids usage^#^**	174 (92.1)	35 (92.1)	118 (90.8)	21 (100)	0.490	0.219
**Follow-up, median (IQR), d**	326 (219-466)	377 (240-449)	325 (219-467)	257 (192-430)	0.475	0.297
**HCMV reactivation within 30d posttransplant**	27 (14.3)	/	24 (18.5)	3 (14.3)	/	0.768
**Time to HCMV reactivation posttransplant, median (IQR), d**	37 (32-43)	/	37 (32-43)	37 (33-44)	/	0.790
**Time to engraftment, median (IQR), d**						
Neutrophil	12 (11-14)	12 (12-14)	12 (11-14)	12 (11-14)	0.578	0.724
Platelet	13 (11-16)	13 (11-16)	13 (11-15)	14 (12-16)	0.666	0.414
**Initial HCMV DNAemia, median (IQR), copies/mL**	2350 (1195-6250)	/	2290 (1192.5-6150)	2760 (1340-14100)	/	0.536
**Peak HCMV DNAemia, median (IQR), copies/mL**	14900 (3555-86800)	/	14450 (3240-86900)	15200 (5300-79400)	/	0.547

**^#^** Recipients received methylprednisolone injection within 30 days after allo-HSCT. **^$^** P value for comparison between three groups by using Kruskal-Wallis test and chi-square or Fisher’s exact test. **^&^** P value for comparison between OCR and RCR groups by using Mann-Whitney U test and chi-square or Fisher’s exact test. * statistically significant P values (P < 0.05). Data are shown as n (%) or median (25th–75th percentile). HCMV, human cytomegalovirus; IQR, interquartile range; AraC, cytosine arabinoside; BuCy, busulfan and cyclophosphamide; MECCNU, methyl cyclohexyl nitrosourea; ATG, anti-human thymocyte globulin; VP-16, etoposide; Flu, fludarabine; BU, busulfan; CLAG, cladribine; DAC, decitabine; MTX, methotrexate; CsA, cyclosporin A; MMF, mycophenolate mofetil; aGVHD, acute graft-versus-host disease; MDS/MPN, myelodysplastic syndrome/myeloproliferative neoplasm; NCR, recipients without HCMV reactivation; OCR, recipients with one episode of HCMV reactivation; RCR, recipients with recurrent HCMV reactivation;/: not applicable.

### HCMV clinical outcomes

All recipients were HCMV-seropositive before allo-HSCT. As shown in **Table 1**, the incidence of acute graft-versus-host disease (aGVHD) was significantly lower in the NCR group, as were the rates of anti-human thymocyte globulin (ATG) and mycophenolate mofetil (MMF) use. The differences in conditioning regimen were attributed to ATG use. However, the NCR group contained more recipients of total-match related donors than did the OCR and RCR groups. There were no significant differences in the above-mentioned characteristics between the OCR and RCR groups, including the magnitude of initial or peak HCMV DNAemia and the time to neutrophil or platelet engraftment.

### Biomarkers associated with HCMV reactivation

**Figure 2:**
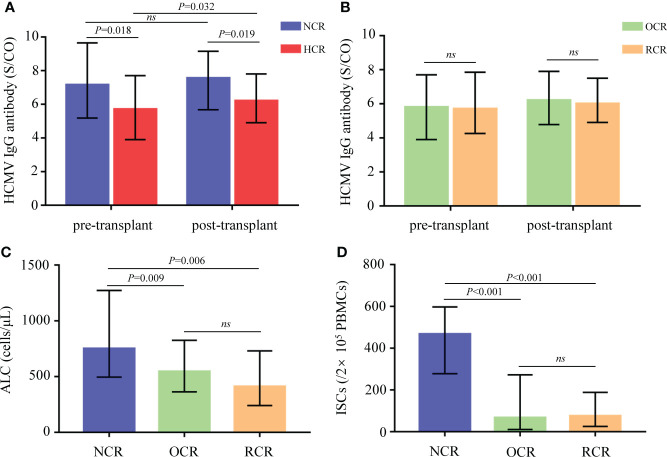
Biomarkers between different groups of recipients. **(A)** Comparisons of pre- and post-transplant HCMV IgG antibody concentrations in recipients from the NCR and HCR groups. **(B)** Comparisons of pre- and post-transplant HCMV IgG antibody concentrations in recipients from the OCR and RCR groups. **(C, D)** Comparisons of ALC and ISCs in recipients from the NCR, OCR and RCR groups. Medians with IQR are shown for each group. ALC, absolute lymphocyte count; ISCs, HCMV specific IFN-γ secreting cells; NCR, recipients without HCMV reactivation; HCR, recipients with HCMV reactivation; OCR, recipients with one episode of HCMV reactivation; RCR, recipients with recurrent HCMV reactivation; ns, no significance.

In total, 189 serum specimens were obtained from recipients within 1 week before and after allo-HSCT. All of the recipients were negative for HCMV IgM (data not shown). Compared to recipients with HCMV reactivation after allo-HSCT in the HCR group, those in the NCR group had significantly higher pre- and post-transplant HCMV IgG concentrations (P = 0.018 and P = 0.019, **Figure 2A**). Moreover, HCMV IgG concentration was significantly elevated after allo-HSCT in recipients experienced HCMV reactivation (P = 0.032), but not in those without HCMV reactivation (**Figure 2A**).

At 30 days after allo-HSCT, recipients who did not experience HCMV reactivation had significantly higher ALC values compared to those who did experience HCMV reactivation (P = 0.009 for the NCR group vs. the OCR group; P = 0.006 for the NCR group vs. the RCR group; **Figure 2C**).

To assess IFN-γ secretion, 83 PBMC samples taken at 30 days posttransplant were stimulated with a pp65 peptide pool. The numbers of ISCs differed significantly according to HCMV outcomes (P < 0.001 for the NCR group vs. the OCR group; P < 0.001 for the NCR group vs. the RCR group; **Figure 2D**).

No significant differences were observed between OCRs and RCRs in HCMV IgG, ALC, or ISCs (**Figure 2**).

The cut-offs of pre- and post-transplant HCMV IgG concentrations, ALC and ISC were 4.75 S/CO, 6.45 S/CO, 865 cells/μL and 190/2×10^5^ PBMCs respectively. In univariate analyses, ATG use, aGVHD, match-related donors, MMF use, and the biomarkers were associated with HCMV reactivation (**Table 2**). Due to the discrepancy in numbers of specimens, different immune indices were analyzed respectively in multivariate analysis. The results showed that ISCs and pre-transplant and post-transplant HCMV IgG concentrations remained significantly associated with the risk for HCMV reactivation, particularly the ISC levels (HR = 0.216, 95% CI 0.112–0.419, P < 0.001; **Table 2**).

**Table 2:** Univariable and multivariate analysis of factors affecting HCMV reactivation.

Variables	HR	95% CI	*P* value
ATG use	3.717	2.054-6.730	<0.001*
aGVHD	1.606	1.138-2.268	0.007*
Total-match related donor	0.239	0.132-0.432	<0.001*
MMF use	6.492	2.065-20.411	0.001*
Pre-transplant HCMV IgG antibody	0.589	0.423-0.821	0.002*
< 4.75 S/CO			
≥ 4.75 S/CO			
Post-transplant HCMV IgG antibody	0.631	0.457-0.871	0.005*
< 6.45 S/CO			
≥ 6.45 S/CO			
ALC	0.593	0.385-0.915	0.018*
< 865 cells/μL			
≥ 865 cells/μL			
ISCs	0.269	0.145-0.501	<0.001*
< 190/2×10^5^ PBMCs			
≥ 190/2×10^5^ PBMCs			
aGVHD			
ATG			
Total-match related donor			
MMF	3.484	1.044-11.622	0.042*
Pre-transplant HCMV IgG antibody	0.665	0.477-0.929	0.017*
aGVHD			
ATG			
Total-match related donor			
MMF			
Post-transplant HCMV IgG antibody	0.697	0.505-0.964	0.029*
aGVHD			
ATG			
Total-match related donor	0.444	0.218-0.903	0.025*
MMF			
ALC			
aGVHD			
ATG			
Total-match related donor			
MMF			
ISCs	0.216	0.112-0.419	<0.001*

*Statistically significant P values (P < 0.05). The cutoffs of biomarkers were determined by X-tile software. Immune indices were analyzed respectively in multivariate analysis. HR and 95% CI were not shown when P value > 0.05. ATG, anti-human thymocyte globulin; aGVHD, acute graft-versus-host disease; MMF, mycophenolate mofetil; ALC, absolute lymphocyte count; ISCs, HCMV specific IFN-γ secreting cells; HR, hazard ratio; 95% CI, 95% confidence interval.

**Figure 3:**
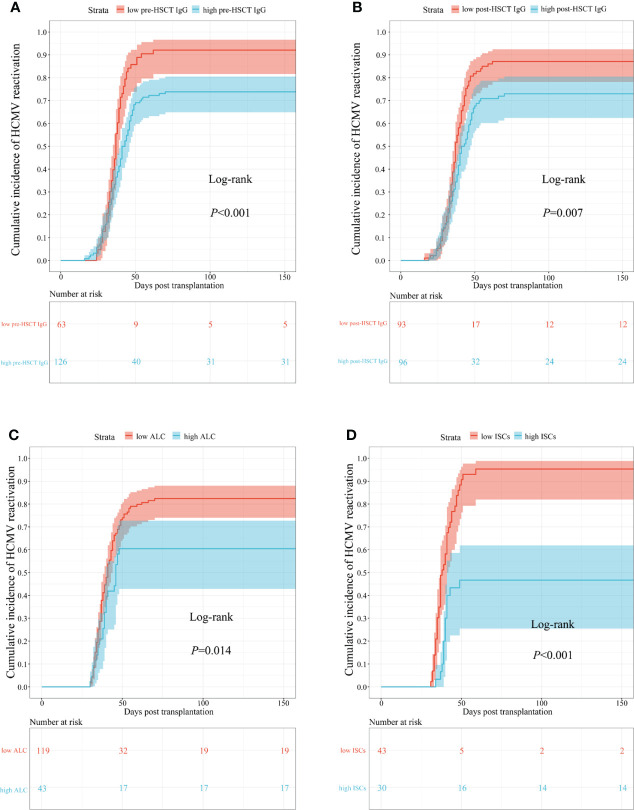
Cumulative incidence curves of time to occur HCMV reactivation in different biomarkers. **(A)** pre-transplant; **(B)** post-transplant HCMV IgG antibody concentrations; **(C)** ALC; **(D)** ISCs. HSCT, hematopoietic stem cell transplant; HCMV, human cytomegalovirus; ALC, absolute lymphocyte count; ISCs, HCMV specific IFN-γ secreting cells.

Relatively low pre-transplant HCMV IgG concentrations were associated with an increased cumulative incidence of HCMV reactivation during the first 150 days (log-rank P < 0.001; **Figure 3A**). The same was true for post-transplant HCMV IgG concentrations (87% vs. 75%; log-rank P = 0.007; **Figure 3B**), ALC (84% vs. 60%; log-rank P = 0.014; **Figure 3C**), and ISC (95% vs. 53%; log-rank P < 0.001; **Figure 3D**).

### Biomarkers associated with the severity of HCMV reactivation

**Figure 4:**
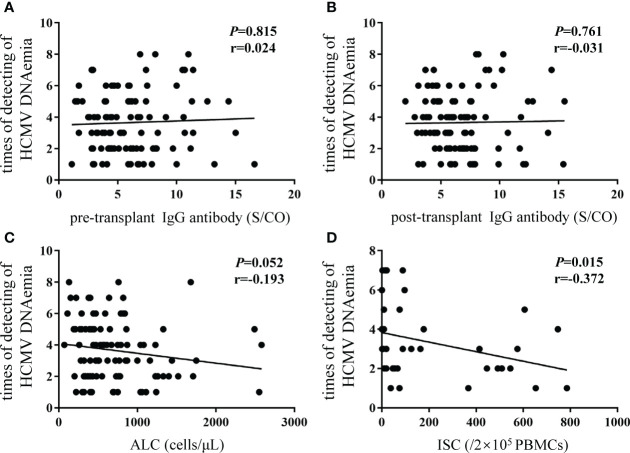
Correlations between duration of HCMV DNAemia and different biomarkers. Y-axis showed the times of continuously detecting HCMV DNA load every week representing the duration of HCMV DNAemia in recipients. **(A)** pre-transplant; **(B)** post-transplant HCMV IgG antibody concentrations; **(C)** ALC; **(D)** ISCs. The P value indicates level of significance and r, the correlation coefficient. HCMV, human cytomegalovirus; ALC, absolute lymphocyte count; ISCs, HCMV specific IFN-γ secreting cells.

The duration of HCMV DNAemia in 102 subjects (in the OCR group) was obtained. No correlations were found between the duration of HCMV DNAemia and pre- or post-transplant IgG concentrations (**Figures 4A, B**). However, there was negative correlation between ALCs and the duration of HCMV DNAemia, with marginal significance (P = 0.052, r = -0.193; **Figure 4C**). The duration of HCMV DNAemia shortened as the number of HCMV-specific ISCs increased (P = 0.015, r = -0.372; **Figure 4D**).

**Figure 5:**
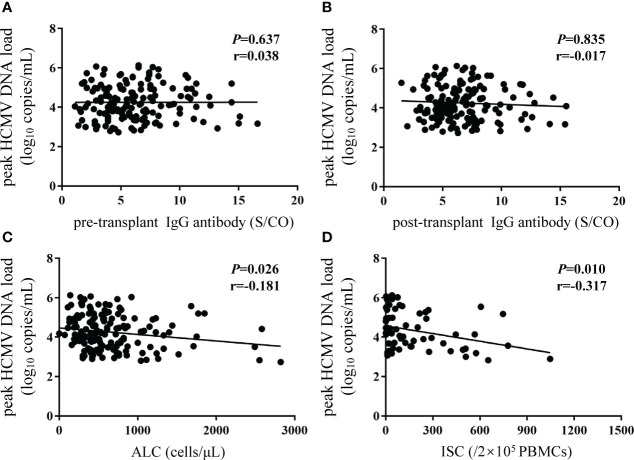
Correlations between peak HCMV DNA load and different biomarkers. Y-axis corresponds to log10 scale. The peak HCMV DNA load was defined as the highest HCMV DNA level recorded after transplantation. **(A, B)** pre- and post-transplant HCMV IgG antibody concentrations; **(C)** ALC; **(D)** ISCs. The P value indicates level of significance and r, the correlation coefficient. HCMV, human cytomegalovirus; ALC, absolute lymphocyte count; ISCs, HCMV specific IFN-γ secreting cells.

The peak HCMV DNA load was not linearly correlated with pre- or post-transplant IgG concentrations (**Figures 5A, B**). However, negative correlations were observed between the peak HCMV DNA load and the ALC (P = 0.026, r = -0.181; **Figure 5C**) and HCMV-specific ISCs (P = 0.010, r = -0.317; **Figure 5D**) in recipients who experienced HCMV DNAemia.

**Figure 6:**
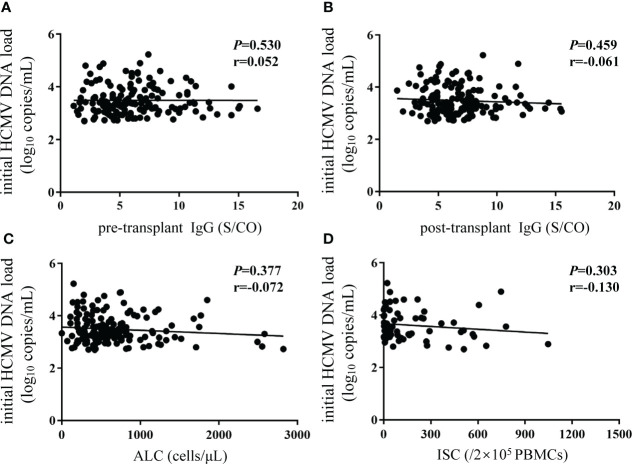
No correlations were observed between initial HCMV DNA load and biomarkers. Y-axis corresponds to log10 scale. The initial HCMV DNA load was defined as the first detectable DNA load after transplantation. **(A, B)** pre- and post-transplant HCMV IgG antibody concentrations; **(C)** ALC; **(D)** ISCs. The P value indicates level of significance and r, the correlation coefficient. HCMV, human cytomegalovirus; ALC, absolute lymphocyte count; ISCs, HCMV specific IFN-γ secreting cells.

No associations were observed between initial HCMV DNA load and HCMV IgG, ALC, or ISCs (**Figure 6**).

## Discussion

Bone marrow transplantation is performed to treat a variety of hematological diseases (Garcia-Manero et al., 2020; Zain and Hanona, 2021), but it causes immunosuppression and promotes opportunistic infections, including HCMV, the most fatal (Ljungman et al., 2011). Antiviral agents (Hodowanec et al., 2020) are used to suppress HCMV reactivation, but this has disadvantages (Koval, 2018). Therefore, a balance between antiviral use and HCMV reactivation needs to be achieved. We evaluated the posttransplant HCMV outcomes of recipients.

Several immunologic factors, including helper and killer T lymphocytes as well as B lymphocytes, are involved in the response to HCMV (Ozdemir et al., 2002; Martins et al., 2019). The ALC is a simple, inexpensive, and readily available parameter that can be monitored frequently after transplantation and reflects the immunologic response to opportunistic infections. In this study, a relatively low ALC was related to more serious HCMV outcomes. This is in agreement with a previous study that found a higher incidence of HCMV infection in a post-transplant lymphopenia group than in a non-lymphopenia group (Yoon et al., 2021). HCMV IgG is typically assayed to confirm previous infection (Torii et al., 2019) and reflects the humoral immune response to HCMV reactivation (Kuo et al., 2008). George et al. and Yen-Ling et al. reported that HCMV IgG levels correlated with T-cell receptor αβ diversity, the size and polyfunctionality of the T-cell response, and T-cell differentiation (Wang et al., 2012; Chiu et al., 2016; Yang et al., 2018). Therefore, anti-HCMV IgG antibody provides protection against HCMV infection, and was associated with HCMV outcomes in recipients after allo-HSCT. HCMV IgM is an indicator of primary infection (Perillaud-Dubois et al., 2020). All recipients were negative for HCMV IgM, so the index was not included in this study. Hence, HCMV IgM was not analyzed in this study. HCMV strain- or glycoprotein complex-specific neutralizing antibodies modulate HCMV infection by preventing binding of HCMV to target cells (Martins et al., 2019; Shibamura et al., 2020). However, the method is complicated and has yet to be standardized. IFN-γ is the most important cytokine in antiviral immunity (Paez-Vega et al., 2018; Wu et al., 2020), and secretion of IFN-γ by CD8+ T cells has been correlated with HCMV reactivation (Liu et al., 2013). In this study, we enumerated all of the cytotoxic cells secreting IFN-γ stimulated by the HCMV pp65 peptide pool and found protective effects posttransplant. Camargo et al. (Camargo et al., 2018) reported that a higher peak HCMV DNA load was related to a lower likelihood of spontaneous clearance, consistent with our findings. Several previous studies (Munoz-Cobo et al., 2011; Gimenez et al., 2014) showed that initial HCMV DNA load had no correlation with the HCMV outcomes or contribution to clinical decisions of preemptive treatment, which may interpret that early immune response was not associated with initial HCMV DNA load in this study.

We excluded related factors from recipient enrollment and the data analysis. In addition, the cutoff values for the immune indices were obtained using X-tile software, which were predictive of the outcomes of HCMV infection, because it was difficult for ROC curves in this data type. There were no significant differences in the cumulative doses of antiviral agents and corticosteroids among different groups, which did not affect the results.

Our study has some limitations. This is a cross-sectional study, so we cannot establish causal relationship between these indicators and HCMV outcomes. Nevertheless, the purpose of our study was to find easy and reliable methods contributed to early judgement of CMV outcomes posttransplant. In the time-to-event and regression analyses, recipients who CMV reactivation occurred previous to the occasion of specimens and data collection were eliminated in order to reasonably assess the value of these biomarkers. Due to the difficulty of collecting specimens at 30 days posttransplant and the lack of lymphocyte at early stage, the obtained specimens about ISCs was less than IgG antibody and lymphocyte. But we analyzed the predictive value respectively. Additionally, the duration of HCMV DNAemia was uncertain for some subjects, resulting in sample sizes of the same immune indices were discrepant in different analyses. We did not obtain the results of HLA typing from participants and stimulate PBMCs with corresponding HLA-restricted peptide. However, the results of ISCs stimulated by overlapping peptide pool of pp65 protein were close to *in vivo* conditions, and it can apply to larger group without regard to HLA typing. The count of lymphocyte and ISCs were performed on 30 days posttransplant, so it is likely that these results were a result of early exposure to CMV; then, we will evaluate earlier time points on samples to assess whether similar results can obtain.

In conclusion, we identified early humoral and cellular immune indices predictive of HCMV outcomes after allo-HSCT, which may be useful for the clinical diagnosis and treatment of HCMV infection.

## Data availability statement

The raw data supporting the conclusions of this article are available upon request from the corresponding author.

## Ethics statement

The studies involving human participants were reviewed and approved by the Ethics Committee of the First Affiliated Hospital, College of Medicine, Zhejiang University. The patients/participants provided their written informed consent to participate in this study.

## Author contributions

JX and JF contributed to the conception and design of the study, GG performed the HCMV DNA detection. SG performed the HCMV IgG and IgM experiments. JX and XL performed the remaining assays. JW and YS performed the statistical analyses. JX wrote the first draft of the manuscript and JF revised the manuscript. All authors approved the final version and take responsibility for the content.

## Conflict of interest

The authors declare that the research was conducted in the absence of any commercial or financial relationships that could be construed as a potential conflict of interest.

## Publisher’s note

All claims expressed in this article are solely those of the authors and do not necessarily represent those of their affiliated organizations, or those of the publisher, the editors and the reviewers. Any product that may be evaluated in this article, or claim that may be made by its manufacturer, is not guaranteed or endorsed by the publisher.
